# Puerarin Inhibits Ferroptosis and Inflammation of Lung Injury Caused by Sepsis in LPS Induced Lung Epithelial Cells

**DOI:** 10.3389/fped.2021.706327

**Published:** 2021-08-04

**Authors:** Baiye Xu, Haidao Wang, Zhen Chen

**Affiliations:** Department of Pediatrics, The First Hospital of Quanzhou Affiliated to Fujian Medical University, Quanzhou, China

**Keywords:** Puerarin, children sepsis, lung injury, ferroptosis, inflammation

## Abstract

**Background:** Ferroptosis is a new type of programmed cell death, which plays an important role in lung injury caused by sepsis. Studies have reported that Puerarin (Pue) can treat lung injury caused by sepsis in children, but whether it plays a role by regulating iron death has not been reported.

**Methods:** LPS induced human alveolar epithelial cell A549 to form a model of lung injury caused by sepsis. MTT detected the effect of Pue on A549 cell viability and the effect of Pue on LPS-induced A549 cell viability. The effects of Pue on LPS-induced inflammatory cytokines TNF-α, IL-8, IL-1β in A549 cells were determined by ELISA assay. The expression level of MDA was detected by TBARS colorimetric quantitative detection kit. GSH kit was used to detect the expression of GSH in cells. The iron kit detected the total iron level and the expression level of ferric divalent ions in the cells. DCFH-DA fluorescent probe was used to detect ROS levels. Western blot was used to detect the expression of ferroptosis-related proteins in cells.

**Results:** Pue alleviated LPS-induced injury and inflammatory response in A549 cells, and Pue reduced the expression of ROS, MDA and GSH in LPS-induced A549 cells. In addition, Pue reduced total iron levels and ferrous ion levels in LPS-induced A549 cells, and decreased the expression of iron ferroptosis-related proteins.

**Conclusion:** Puerarin inhibited ferroptosis and inflammation of lung injury caused by sepsis in children in LPS induced lung epithelial cells.

## Introduction

Sepsis in children is one of the important causes of death in children (1). Sepsis in children is a systemic inflammatory response syndrome caused by infection, often causing damage to multiple organs and systems. The main clinical symptoms are the disorder of the immune system and the uncontrolled inflammatory response, which eventually leads to dysfunction of all organs and even death in severe cases (2, 3).

Study (4) suggests that the target organ more easily involved in sepsis is lung tissue. The main pathology is that in sepsis, a large number of inflammatory mediators and lipid metabolites enter the blood circulation, which stimulates the accumulation and activation of inflammatory cells in the lung tissue of patients, thus producing more chemokines and oxygen free radical cytokines, which aggravate the inflammatory response and form a chain reaction (5, 6). At this point, the balance between anti-inflammatory and pro-inflammatory mediators cannot be reached, resulting in anti-inflammatory response syndrome, thus resulting in damage to capillary endothelial cells and alveolar epithelial cells in lung tissue, increased permeability of pulmonary capillaries to protein, obstruction of fluid exchange between blood vessels and vascular interstitium in lung tissue, and ultimately leading to the occurrence of permeable pulmonary edema (7–9). If not treated promptly and effectively, acute lung injury often develops into acute respiratory distress syndrome.

Ferroptosis is a new type of programmed cell death, which is different from apoptosis, necrosis and autophagy (10). Under the action of divalent iron or ester oxygenase, the unsaturated fatty acids which are highly expressed on the cell membrane are catalyzed to produce liposome peroxidation, which induces cell death (11). In addition, the expression of antioxidant systems, including glutathione (GSH) and glutathione peroxidase 4 (GPX4) was decreased, while GPX4 had the function of reducing liposome peroxidation and repairing the oxidative damage of membrane lipids (12–14). GPX4 plays a protective role in bacterial infection and multibacterial sepsis, and as an essential channel for iron death and coke death, GPX4 may be an important molecular target for the development of effective drugs for infection and sepsis (15, 16). The main manifestation of ferroptosis in cells is the increase of lipid peroxidation and ROS (17). Study has shown that imbalance of body oxides and antioxidants plays an important role in lung injury caused by sepsis (5). Therefore, ferroptosis plays an important role in sepsis- induced acute lung injury.

Puerarin (Pue), a flavonoid monomer, has been widely used in the treatment of cardiovascular disease, liver disease and diabetes mellitus due to its beneficial effects in anti-inflammatory, antioxidant and anti-atherosclerosis aspects (18–20). Its mechanism of action is related to apoptosis and antioxidant stress. At the animal level, Pue can improve LPS-induced lung injury by inhibiting inflammatory response (21). However, the effect of Pue on ferroptosis in sepsis-induced lung injury has not been reported.

In this paper, we tested the effect of Pue on ferroptosis and inflammatory response in lung epithelial cells induced by LPS, so as to provide a theoretical basis for Pue treatment of lung injury.

## Materials and Methods

### Cell Culture and Treatment

We used 10 μg/mL lipopolysaccharide (LPS) to simulate Human alveolar type II epithelial cells A549 cells for 16 h to simulate sepsis-induced lung injury (22). A549 cell line was purchased from the Shanghai Cell Collection (Shanghai, China), and cells were cultured in Dulbecco's modified Eagle's medium (DMEM; Gibco; Thermo Fisher Scientific, Inc.) supplemented with 10% fetal bovine serum (FBS; Gibco; Thermo Fisher Scientific, Inc.) at 37°C in a humidified atmosphere of 5% CO_2_.

### MTT Assay

A549 were plated in 96 well plates (1 ×10^6^ cells/mL) for12 h. After Puerarin treatment, MTT (5 mg/mL in PBS, 10 μL, Thermo fisher scientific, Rockford, IL, USA) was added to each well and incubated at 37°C for 3 h in the dark. After incubation, the culture medium was replaced with 100 μL DMSO, and the absorbance was quantitated at 570 nm using a multi-well spectrophotometer (Molecular Devices, Sunnyvale, CA, USA).

### ELISA Assay

TNF-α ELISA kits (ab181421, Abcam, UK), IL-8 ELISA kit (ab46032, Abcam, UK) and IL-1β ELISA kit (ab214025, Abcam, UK) were used to measure the cell TNF-α, IL-8 and IL-1βfollowing the instructions.

### Lipid Peroxidation Assay

GSH enzyme activity was measured with a GSH-Px Assay Kit (Cayman Chemical, Ann Arbor, MI, USA), which is commercially available. It was used in accordance with the manufacturer's protocol. Malondialdehyde (MDA) activity was measured with TBARS quantitative kit (C10445, Thermo fisher scientific, USA) according to the manufacturer's protocol.

### Iron Concentration Detection

To detect iron concentration in the cells during sepsis, an iron assay kit (MAK025, Sigma-Aldrich) was used according to the manufacturer's protocol.

### Fe^2+^ Assay

To detect Fe^2+^ content in the cells, an iron assay kit (ab83366, Abcam, UK) and presented as nanogram Fe^2+^ per milligram of protein according to the manufacturer's protocol.

### ROS Assay

ROS levels of cells were detected using a fluorescent probe, 2′,7′-dichlorodihydrofluorescein (DCHF) (Sigma), which could be rapidly oxidized into the highly fluorescent 2′,7′-dichlorofluorescein (DCF) in the presence of intracellular reactive oxygen species (ROS). Fluorescence was monitored with a laser scanning confocal microscope (Leica, Germany) at 488 nm. The amount of ROS was quantified as the relative fluorescence intensity of DCF per cell in the scan area.

### Western Blot

A549 cells were collected and lysed with RIPA lysis buffer (Beyotime Institute of Biotechnology) at 4°C for 30 min. Then proteins were detected using a BCA protein assay kit (Bio-Rad Laboratories, Inc.). Loading buffer was added to cytosolic extracts, and after boiling for about 5 min, 30 μg of protein of each sample were resolved by 10% sodium dodecyl sulfate-polyacrylamide gel electrophoresis (SDS-PAGE), and then the total gel was transferred into polyvinylidene fluoride (PVDF) membranes. The membranes were blocked with 10% skimmed milk for 2 h at room temperature, followed by incubation with anti- SLC7A11 (ab175186, Abcam, UK), anti- GPX4 (ab125066, Abcam, UK), anti- FTH1 (ab75972, Abcam, UK), anti- NOX1 (ab78016, Abcam, UK) and anti-GAPDH (ab8245, Abcam, UK) primary antibodies overnight at 4°C with 1: 1,000 dilution followed by incubation with horseradish peroxidase-conjugated secondary antibodies (goat anti-rabbit IgG,1:5,000, ab172130, Abcam). The signals were detected using enhanced chemiluminescence reagent (GE Healthcare) and Image J software (version 146; National Institutes of Health, Bethesda, MD, USA) was used to analyze the fold-changes of protein levels.

### Statistical Analysis

All data were analyzed with the GraphPad Prism 7.0 software (GraphPad Software, Inc.). Comparisons among multiple groups were analyzed using one-way ANOVA followed by Tukey's *post hoc* test. *P* < 0.05 was considered to indicate a statistically significant difference. The data in this study are expressed as the mean ± standard deviation (SD). All experiments were repeated three times independently.

## Results

### Pue Alleviated Cell Injury of LPS-Induced A549 Cells

**Figure 1:**
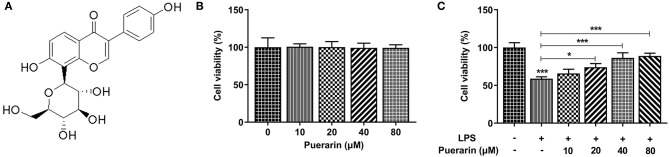
Pue alleviated cell injury of LPS-induced A549 cells. **(A)** The chemical formula of Pue. **(B)** MTT detected the cell viability when different condition of Pue induced. **(C)** MTT detected the cell viability in LPS-induced cells when different condition of Pue induced. *N* = 5. *P < 0.05 and ***P < 0.001.

The chemical formula of Pue is shown in Figure 1A. After Pue of different concentrations (0, 10, 20, 40, and 80 μM) was applied to A549 cells, cell viability was measured by MTT. The results were shown in Figure 1B, indicating that cell viability was not affected within this concentration range. A549 cells were induced by 10 μg/mL LPS for 16 h, and then Pue of different concentrations was applied to A549 cells. It was found that the viability of A549 cells induced by LPS was significantly increased with the increase concentration of Pue (Figure 1C). The results showed that Pue could enhance activity of LPS-induced A549 cell.

### Pue Alleviated Inflammation of LPS-Induced A549 Cells

**Figure 2:**
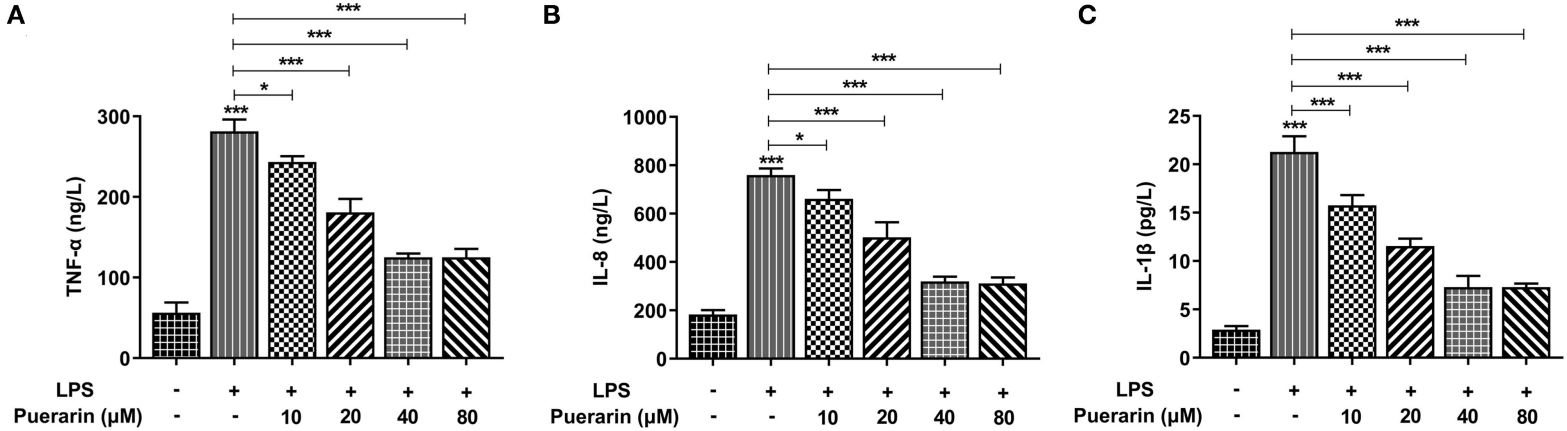
Pue alleviated inflammation of LPS-induced A549 cells. ELISA assay detected the expression of TNF-α **(A)**, IL-8 **(B)**, and IL-1β **(C)** in LPS-induced cells when different condition of Pue induced. *N* = 5. *P < 0.05 and ***P < 0.001.

We detected the expression of inflammatory cytokines TNF-α (Figure 2A), IL-8 (Figure 2B), and IL-1β in A549 cells, and they increased significantly in LPS-induced A549 cells (Figure 2C), while Pue decreased significantly with increased Pue concentration, indicating that Pue alleviated the LPS-induced inflammatory response in A549 cells.

### Pue Alleviated Lipid Peroxidation of LPS-Induced A549 Cells

**Figure 3:**
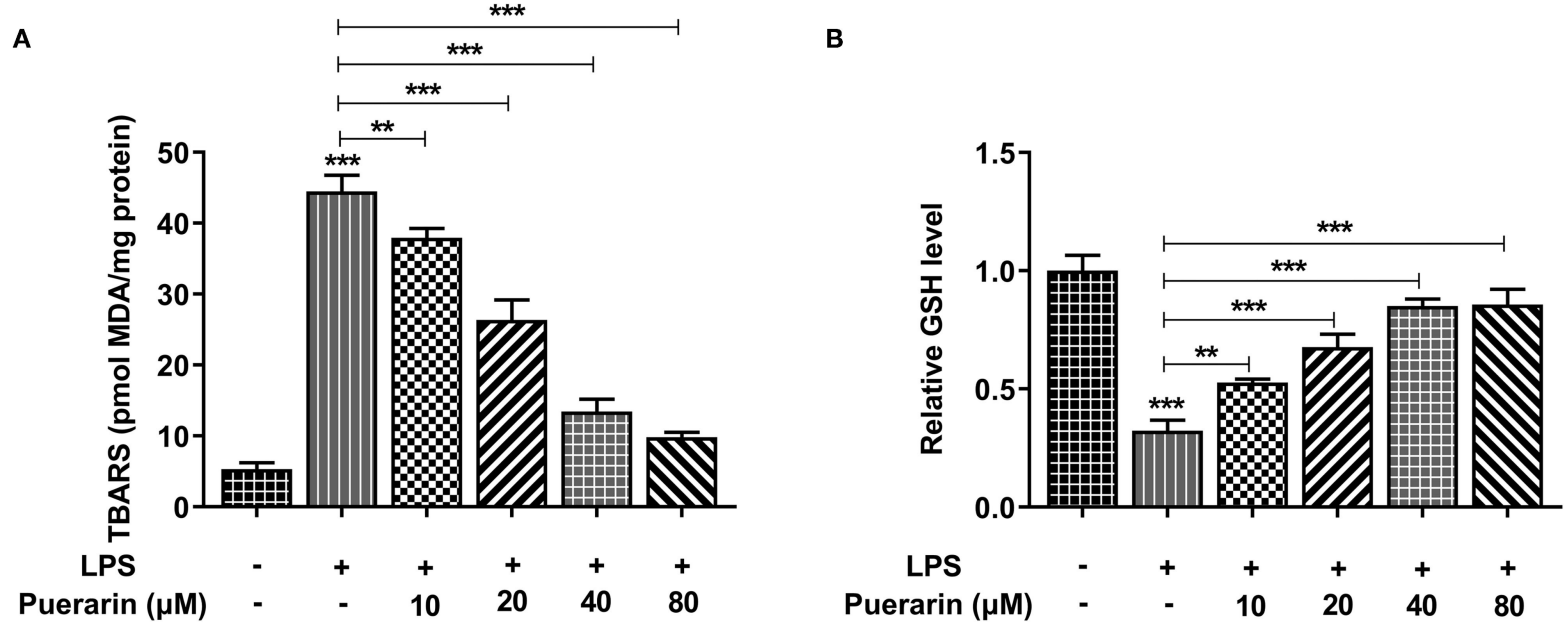
Pue alleviated lipid peroxidation of LPS-induced A549 cells. **(A)** The TBARS production rate was detected by TBARS quantitative kit in LPS-induced cells when different condition of Pue induced. **(B)** The GSH production rate was detected by GSH quantitative kit in LPS-induced cells when different condition of Pue induced. *N* = 5. **P < 0.01 and ***P < 0.001.

We used a kit to detect the production rate of TBARS to determine the expression of lipid peroxide MDA, and used an ELISA kit to detect the expression of GSH. We found that compared with the control group, the expression of MDA increased after LPS induction (Figure 3A), while the expression of GSH decreased (Figure 3B). After Pue was applied to LPS-induced A549 cells, MDA expression gradually decreased and GSH expression gradually increased with the increase of Pue concentration. The results showed that Pue could reduce LPS-induced lipid peroxidation in A549 cells.

### Pue Inhibited Ferroptosis of LPS-Induced A549 Cells

**Figure 4:**
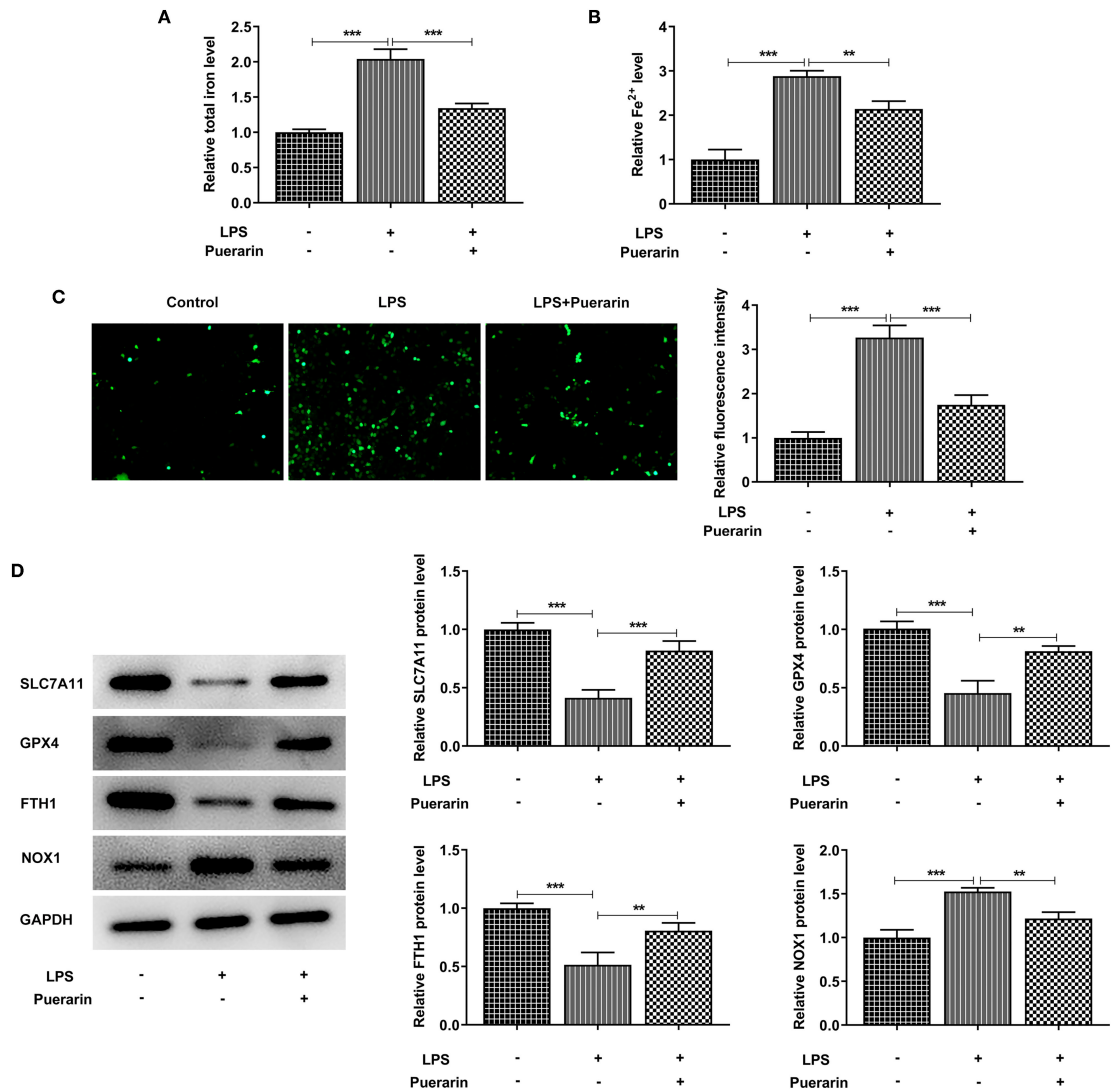
Pue inhibited ferroptosis of LPS-induced A549 cells. **(A)** Iron Assay Kit detected total iron levels in cells in LPS-induced cells when Pue induced. **(B)** Iron Assay Kit detected Fe^2+^ levels in cells in LPS-induced cells when Pue induced. **(C)** ROS levels were detected with DCFH-DA fluorescent probe in LPS-induced cells when Pue induced. *N* = 5. **(D)** The expression of iron death related proteins was detected by Western blot in LPS-induced cells when Pue induced. *N* = 3. **P < 0.01 and ***P < 0.001.

Through the above experiments, Pue with concentration of 80 μM was selected for subsequent experiments. We found that after LPS induction, the total iron level in the cells was significantly increased compared with the control group (Figure 4A), and the expression of ferric divalent ions (Figure 4B) was also significantly increased. After Pue treatment, the expression of total iron and divalent iron decreased significantly compared with that of LPS group. Subsequently, the expression level of ROS was detected by DCFH-DA fluorescent probe. As shown in Figure 4C, Pue can significantly reduce the expression level of ROS in LPS-induced A549 cells. Western blot was used to detect the expression of ferroptosis related proteins SLC7A11, GPX4, FTH1, and NOX1 in cells. Compared with the control group, SLC7A11, GPX4, and FTH1 expression in the LPS group decreased, while NOX1 expression increased. Compared with the LPS group, the expression of SLC7A11, GPX4, and FTH1 was increased and the expression of NOX1 was inhibited after Pue was applied to the cells (Figure 4D). The results showed that Pue could inhibit the ferroptosis of A549 cells induced by LPS.

## Discussion

Impair of lung function is the most common complication of sepsis in children, mainly manifested as acute lung injury, which has a high mortality rate (23). LPS is the main component of the cell wall of Gram-negative bacteria. Endotoxemia and systemic inflammatory response syndrome caused by Gram-negative bacteria infection are the main causes of acute lung injury in sepsis in children (24). Currently, LPS-induced lung injury of sepsis models are very mature, and the authenticity and reliability of LPS-induced septic lung injury have been confirmed (25, 26). In our study, it was found that after LPS induced A549 cells, cell viability decreased significantly, inflammatory response increased significantly, and lipid peroxidation occurred in the cells, indicating the success of model induction.

Dixon et al. (27) discovered ferroptosis, a new type of cell death, during a high-throughput screening of ferroptosis inducers in 2012. Ferroptosis is a cell death mode characterized by iron overload and peroxide accumulation. Its morphology is mainly manifested as cell membrane rupture, absence of chromosome condensation, increase of mitochondrial membrane density and decrease of mitochondrial membrane volume (10, 28). Existing studies have shown that ferroptosis has an important effect on the pathological process of many diseases, such as tumors (29), neurosystemic diseases (30), ischemia reperfusion injury (31), kidney injury (32), etc. Sepsis, acute lung injury, acute kidney injury and other critical diseases are characterized by inflammation and oxidative stress, followed by lipid peroxidation (33). Study has shown that folate-induced lipid peroxidation and glutathione metabolic protein down-regulation in mice with acute kidney injury are typical features of ferroptosis. The ferroptosis inhibitor improved renal function and reduced histological damage in mice (34). In addition, the inactivated ferritin regulator GPX4 can cause acute renal failure in mice (35). Liu et al. reported that Ferrostatin-1, an ferroptosis inhibitor, is capable of treating LPS—induced acute lung injury, and ferroptosis may be a new therapeutic target for patients with acute lung injury (22).

Pue, a proven antioxidant and anti-inflammatory monomer in Traditional Chinese medicine, has been widely used in clinical treatment of cardiovascular and cerebrovascular diseases and diabetic nephropathy (36–38). It has been reported that Pue can inhibit inflammatory response to prevent LPS- induced acute lung injury (21). In our study, Pue was found to reduce LPS-induced damage to A549 cells and reduce the expression of inflammatory cytokines TNF-α, IL-8, and IL-1β in LPS-induced A549 cells, thereby inhibiting the inflammatory response. At present, only Liu et al. reported that Pue can prevent heart failure caused by stress load by reducing ferroptosis (39). However, whether Pue plays a role in ferroptosis in sepsis in children-induced lung injury has not been reported.

The main mechanism of ferroptosis is that under the action of iron divalent or lipoxygenase, the unsaturated fatty acids highly expressed on the cell membrane undergo liposome peroxidation and produce ROS, leading to cell death. Excess iron promotes the production of superoxide and leads to lipid peroxidation via free radicals in fenton reactive (40). In addition, GSH depletion and GPX4 reduction also lead to the occurrence of ferroptosis (41). Moreover, in mice with acute lung injury, iron overload, GSH depletion, MDA accumulation, and GPX4 and ferritin expression levels in lung tissues were decreased (42). It can be inferred from the above that the changes of iron content, MDA and GSH are related to the death of lipid peroxidation. In our experiment, it was found that after Pue was treated on LPS-induced A549 cells, the expression of total iron and divalent iron decreased significantly, the expression of MDA increased, and the expression of GSH and ROS decreased. In addition, the expression of ferroptosis-related proteins SLC7A11, GPX4, and FTH1 increased, while the expression of NOX1 decreased. These results suggest that Pue improves sepsis-induced lung injury by inhibiting ferroptosis.

## Conclusion

Puerarin can inhibit ferroptosis and inflammation of lung injury caused by sepsis in LPS induced lung epithelial cells. Our paper provided a theoretical basis for the treatment of sepsis in children.

## Data Availability Statement

The raw data supporting the conclusions of this article will be made available by the authors, without undue reservation.

## Author Contributions

BX wrote the manuscript, analyzed the data, and carried out the experiments. HW and ZC supervised the present study, searched the literature, and revised the manuscript. All authors read and approved the final manuscript.

## Conflict of Interest

The authors declare that the research was conducted in the absence of any commercial or financial relationships that could be construed as a potential conflict of interest.

## Publisher's Note

All claims expressed in this article are solely those of the authors and do not necessarily represent those of their affiliated organizations, or those of the publisher, the editors and the reviewers. Any product that may be evaluated in this article, or claim that may be made by its manufacturer, is not guaranteed or endorsed by the publisher.
